# The Roles of Obstetrics Training Skills and Utilisation of Maternity Unit Protocols in Reducing Perinatal Mortality in Limpopo Province, South Africa

**DOI:** 10.3390/healthcare10040662

**Published:** 2022-04-01

**Authors:** Langanani C. Makhado, Mutshinyalo L. Mangena-Netshikweta, Seani A. Mulondo, Foluke C. Olaniyi

**Affiliations:** 1 Department of Advanced Nursing Science, Faculty of Health Sciences, University of Venda, Thohoyandou 0950, South Africa; langananimakhado@gmail.com (L.C.M.); seani.mulondo@univen.ac.za (S.A.M.); 2 Department of Public Health, Faculty of Health Sciences, University of Venda, Thohoyandou 0950, South Africa; foluolaniyi@yahoo.com

**Keywords:** antenatal care, intrapartum care, maternity unit protocol, obstetric skills, perinatal mortality

## Abstract

Perinatal mortality has been associated with poor maternal health during pregnancy and intrapartum periods. This study was conducted to determine the effects of obstetrics training programmes and the utilization of maternal unit protocols in the management of obstetric complications in reducing neonatal mortality rate in selected public hospitals in the Vhembe district of Limpopo province, South Africa. A quantitative, descriptive design was used and a non-probability purposive sampling method was used to select midwives with a minimum of two (2) years of working experience in maternity wards of selected public hospitals. A total of 105 completed questionnaires were analysed using SPSS version 23. Most of the respondents were within the age group of 40–59 years (74.3%) and with professional experience of more than 10 years (76.8%). More than half (63.8%) had qualified as midwives at a diploma level. Only 44.8% indicated that the protocols were always utilised, even though the majority (70.5%) believed that the protocols are helpful in managing obstetrics complications. The obstetric skills are helpful in reducing neonatal mortality, however, utilisation of the protocols is not encouraging in the studied health facilities. We recommend that efforts should be geared towards the enforcement of the protocol’s use, and all midwives should be encouraged to undergo the trainings.

## 1. Introduction

Perinatal mortality is a major public health concern globally, especially in developing countries, where the prevalence of perinatal mortality has been reported to be more than 10-fold greater in comparison with the prevalence in developed countries [1]. Poor perinatal health reflects issues of poor maternal health during the antenatal and intrapartum periods and it influences poor perinatal outcomes such as perinatal morbidity and mortality [2,3]. The most common causes of perinatal mortality rates are maternal hypertensive disorders, infection, asphyxia, pre-term birth and obstetric complications; however, all of these factors are largely avoidable. Perinatal mortality is viewed as a vital indicator of the health of a nation because of its association with the quality of and access to medical care, socio-economic conditions, cultural, household, environmental, biological and public health practices [4,5,6].

According to international standards, perinatal deaths include both stillbirth (deaths from 22 completed weeks of gestation) and neonatal death (from birth to the seventh completed day of life for early neonatal deaths and twenty-eight completed days of life for late neonatal death) [6]. In South Africa, the Births and Deaths Registration Act (Act No. 51 of 1992) defines a stillbirth as a foetus that has at least 26 weeks of intra-uterine existence but shows no sign of life after complete birth [7].

Recent estimates show that the perinatal mortality rate in high-income countries of the world is about 10 per 1000 live births compared with 50 per 1000 live births in low-income countries [8]. Globally, it was estimated that more than 2.6 million stillbirths occurred in 2015 during the third trimester period [9]. Furthermore, it is estimated that over 3.3 million infants are born dead every year while 33% of these deaths occur at birth, largely due to avoidable causes. Of all baby deaths, 98% occur in the developing world, with 1.16 million taking place in Sub-Saharan Africa and stillborns account for more than 50% of all perinatal deaths [10,11,12,13,14]. Several deaths were connected to inadequate medical and nursing intervention during birth. Therefore, providing expert care to mothers during pregnancy, as well as during and after birth and in particular in the first 28 days, critically contributes to child survival [5].

Risk determinants for perinatal mortality entail maternal age, race, marital status, parity, birth weight, gestational age at birth, labour complications, rates of antenatal visits, antepartum haemorrhage, parity, hypertension, pre-eclampsia and eclampsia [15,16].

Sub-Saharan African countries have been reported to have the highest number of perinatal deaths globally [2]. In some African countries, there is a high probability of underestimation of perinatal mortality rates due to substantial under-reporting and insufficient reporting structures, particularly for home-based deliveries [17].

Addressing perinatal mortality problems requires multiple intervention strategies as well as adequate infrastructure for obstetric and neonatal care [1]. A randomised controlled trial study involving five low-income countries and a middle-income country reported that the application of community and facility-based intervention programmes involving the improvement of access to quality obstetrics and neonatal care through community mobilization, training of community birth attendants on early identification of problems and staff training on the management of obstetrics and neonatal emergencies, did not result in an expected reduction in perinatal mortality [1]. Moreover, an evaluation of the application of the World Health Organization (WHO) Safe Childbirth Checklist Program in a matched-pair, cluster-randomised controlled trial study in India, using 60 paired facilities showed that the application of the WHO tool did not account for a significant reduction in perinatal mortality [18].

However, a systematic review and meta-analysis study involving 20 trials with 1653 805 births showed that trainings on neonatal resuscitation are significantly associated with reduced perinatal mortality rates [19]. In addition, a systematic review involving seven studies demonstrated that the implementation of Helping Babies Breathe resuscitation techniques resulted in a significant decrease in perinatal mortality [20]. Delivery at a health facility, rather than at home, has also been reported to result in a lower rate of perinatal mortality [21].

To reduce the perinatal mortality rate in South Africa, free reproductive health and maternity health services were introduced, such that pregnant women and young children up to age 5 years can access medical care free of charge. Workshops and in-service trainings were also scheduled for doctors and midwives on national programmes such as the use of Basic Antenatal Care (BANC) protocol for early identification of problems, Essential Steps in Managing Obstetric Emergencies (ESMOE) and Helping Babies Breath (HBB) skills. Furthermore, maternal protocols were drafted to be followed when dealing with maternity health care services to prevent complications. Midwives in public hospitals are expected to observe these protocols when providing services to women in labour, and the protocols are made available in each maternity ward for easy accessibility by midwives.

Through these interventions, the institutional neonatal death rate reduced from 12.7 deaths per 1000 live births in 2016 to 12 deaths per 1000 live births in 2018; however, it has remained at that rate till 2021 [22]. The country still aims to achieve the Sustainable Development Goal (SDG 3.2) of reducing neonatal mortality to less than 12 deaths per 1000 live births by reducing neonatal mortality and stillbirths by 50% by the year 2030 [23]. The purpose of this study was to determine the effects of these training programmes on midwives and the utilization of maternal unit protocols in the management of obstetric complications to further reduce the neonatal mortality rate in selected public hospitals in the Vhembe district of Limpopo province, South Africa.

## 2. Materials and Methods

### 2.1. Study Setting

This study was conducted in the Vhembe District, one of the five districts of Limpopo province of South Africa which covers an area of 25,597 km^2^ and has a total population of 1, 402, 779 (as of 2020) with 54% females and 46% males [24]. The Vhembe district is largely rural with more households headed by females [24] as males usually migrate to the urban areas for employment opportunities. The area is faced with infrastructural backlogs for water, sanitation and electricity which negatively impact the health of the communities. The district is home to seven public hospitals and the number of deliveries recorded in the Vhembe District is estimated to be about 1, 921 per month [25].

### 2.2. Study Design

The study utilised a quantitative, descriptive design to obtain relevant information related to midwives’ training on obstetrics’ skills as well as the availability and utilisation of maternity unit protocols when managing obstetric complications in the selected public hospitals of Vhembe district in Limpopo province, South Africa.

### 2.3. Population and Sampling

Extreme or deviant case sampling, which is a type of purposeful or criterion-based sampling, was used to select three hospitals included in this study. This type of sampling method considers highly unusual manifestations of the phenomenon of interest [26]. The public hospitals in Vhembe District were sorted based on the number of midwives and record of deliveries obtained from existing hospital records (Table 1). Subsequently, the three hospitals with the highest numbers of midwives and deliveries per month were included in the study.

The total population consists of all the midwives in the selected healthcare facilities who were registered with the South African Nursing Council and have at least two years of working experience in maternity wards. Non-probability purposive sampling was used to collect data from the respondents who were readily available, particularly the midwives working in the labour ward [27].

### 2.4. Data Collection Tool

A self-administered questionnaire was used to collect data in this study. The tool consists of a total of 38 questions (both closed-ended and open-ended questions) divided into 5 sections: sociodemographic information of the respondents, exposure of midwives to the training skills (BANC, ESMOE and HBB), attitudes of midwives towards the women in labour, availability and implementation of guidelines/protocols to manage complications and the staffing and workload of the midwives in the health facilities. To assess the level of exposure of midwives to the recommended training skills, close-ended questions such as “Have you been trained on essential steps in managing obstetric emergencies” and “Do you think there is sufficient awareness about HBB training?” were asked, to which the respondents were expected to select a “Yes” or “No” option. Open-ended questions such as “How often do you think the trainings should be conducted” were also included to allow the participants to air their opinions on the current and expected schedule of the training programmes. To determine the attitudes of midwives towards women in labour, close-ended questions were designed in a Likert scale format, where the respondents were expected to select the options that represent their opinions out of five options ranging from “Strongly disagree” to “Strongly agree”. The types of questions asked in this section include: “Midwives encourage and assist the women with breathing and relaxation techniques”; “Midwives want the women to undergo natural labour without pain- relief medication”. To determine the availability of the guidelines and their implementation by midwives, some statements such as: “Are maternal guidelines/protocols readily available or accessible to all midwives in the unit?” and “Do you think all midwives utilise maternal guidelines when managing the obstetric emergencies and complications?” were presented to respondents and they were required to select appropriate responses from 1–5 (1—never; 2—hardly ever; 3—sometimes; 4—often; 5—always).

### 2.5. Data Collection Procedure

Permission to conduct the study was obtained from the University of Venda, the Department of Health, Limpopo Province and the Chief Executive Officers of the selected hospitals. A self-administered questionnaire with closed and open-ended questions was used to obtain information from the midwives about their knowledge of the availability and utilisation of maternal protocols when managing obstetric emergencies. The validity of the instrument was ensured by presenting it to experts in the Department of Advanced Nursing Sciences for their review, and they confirmed that it measures the intended concepts. The questionnaire was also pre-tested with selected midwives at one of the public hospitals which was not included in the main study, to identify and clarify confusing concepts before its administration to the main respondents. However, no items on the instrument were found to be confusing during pre-testing. The researcher (first author) visited the hospitals, booked an appointment with prospective respondents, explained the study to them, obtained their written consent and distributed the questionnaires to them. Then, she allowed the respondents to complete the questionnaires at a time convenient for them, to avoid interference with their professional duties and returned at an appointed time to collect the completed questionnaires. A total of 110 questionnaires was distributed to available midwives in the three selected hospitals and data were collected over a period of 3 months.

### 2.6. Ethical Considerations

Ethical clearance was granted by the University of Venda Research and Ethics Committee (SHS/16/PDC/36/0602). The respondents were assured of anonymity, their rights to participate or withdraw from the study at any time which was explained to them and the principles of voluntary participation, confidentiality and avoidance of harm were maintained throughout the study. Each respondent signed the informed consent form before being given a questionnaire to complete.

### 2.7. Data Analysis

Data were collected, coded and captured using Microsoft Excel 2013. Captured data were then cleaned prior to commencing the data analysis process. Missing data were handled by replacing missing values with their corresponding variable averages. Cleaned data were then analysed using the Social Package for Social Sciences (SPSS Version 23.0). Frequencies distributions were used to give a summary of responses given by the study participants. Descriptive statistics (measures of central tendency and measures of dispersion) were also used to describe and understand the gathered data. Chi-squared tests for association were used to identify hospital and participant characteristic variables that were related to the BANC and ESMOE training skills. Symmetric measures such as the Phi, Cramers’ V and Contingency Coefficient were used to quantify the strength of the identified relationships between variables.

## 3. Results

A total of 105 questionnaires were fit for analysis, making a good response rate of 95%. The distribution of respondents as per hospital is: hospital A (*n* = 47, 44.8%), hospital B (*n* = 22; 20.9%) and hospital C (*n* = 36, 34.3%).

### 3.1. Sociodemographic Characteristics

Most the respondents were females (*n* = 103, 98.1%) within the age group of 40–59 years (*n* = 76, 74.3%). More than half of them (*n* = 67, 63.8%) had qualified as midwives at a diploma level while 24 (22.9%) held degrees in General nursing, Community, Psychiatry, and Midwifery. However, most of them (*n* = 84, 80.0%) did not have any area of specialty, only 18 (17.1%) had a Diploma in Advanced Midwifery and Neonatology. Regarding the number of years of experience, most of the respondents (*n* = 96, 76.8%) have more than 10 years of experience in the nursing profession (Table 2).

### 3.2. Exposure to the Training Skills

A chi-squared test for independence was performed to investigate the level of association between age and whether midwives had been exposed to the BANC skills training programme. The test results showed the existence of relationship between the two variables (chi-square = 29.946, df = 4, *p* < 0.05). Symmetric measures which measure the strength of the relationship were used to conclude that the relationship was a strong one based on statistically significant Cramer’s V and Contingency coefficient values of 0.531 and 0.471, respectively (*p* < 0.05). Similarly, a strong association was found between specialty qualifications and exposure to BANC training (chi-square = 9.015, df = 3, *p* < 0.05; Cramer’s V = 0.321, *p* < 0.05) as well as work experience and BANC training (chi-square = 18.364, df = 3, *p* < 0.05, Cramer’s V = 0.426, *p* < 0.05).

Moreover, chi-squared tests for independence showed that a strong relationship existed between nursing qualifications possessed by midwives and whether midwives had received ESMOE training (chi-square = 15.433, df = 2, *p* < 0.05; Cramer’s V = 0.363, *p* < 0.05). However, the association between specialty qualifications and ESMOE training, though statistically significant, was found to be a weak one (chi-square = 9.527, df = 3, *p* < 0.05; Cramer’s V = 0.287, *p* < 0.05).

Concerning the HBB skills, a relationship was found between nursing qualifications and HBB skills training (chi-square = 4.759, df = 2, *p* < 0.05). However, quantified strength of the relationship showed the association between the two variables was weak following from the computed Cramer’s V value below 0.3 (Cramer’s V = 0.212, *p* < 0.05). Moreover, the relationship between specialty qualifications and HBB training was found to be a weak one (chi-square = 7.388, df = 3, *p* < 0.05; Cramer’s V = 0.239, *p* = 0.118).

### 3.3. Availability and Utilization of Maternity Unit Protocols

While more than half of the respondents (n = 59, 56.2%) confirmed that the protocols were always available, only 47 (44.8%) indicated that the protocols were always utilised. Some of the respondents confessed that the protocols were hardly ever or even never utilised.

### 3.4. Awareness Creation Regarding the Utilisation of Protocols

Many respondents (61%) admitted that awareness of the utilisation of the maternity unit protocol is “always” or “often” created for the nursing staff, and the majority (n = 74, 70.5%) believed that the protocols are helpful in managing obstetrics complications.

### 3.5. Management Support on Utilisation of Protocols

A good number of the respondents (60.9%) indicated that midwives always received assistance from the hospitals’ management with regards to the protocols’ utilisation.

## 4. Discussion

The participants in this study were mostly females, confirming that the nursing profession, especially midwifery, is female dominated. This is not unexpected as the nursing profession has been regarded to be traditionally and predominantly considered to be a female profession [28]. In addition, because of the cultural and religious beliefs of some pregnant women that men should not be allowed in the maternity units, resulting in the discrimination and rejection of male midwives [29], many male midwives prefer to work in any other units in the hospitals than the maternity units. The respondents also consist of more mature professionals within the age group 40–59 years and many years of experience. Thus, they are expected to have a good understanding of the maternity unit protocols and utilise them properly. Unfortunately, only a few of the respondents specialised in specific areas, even though being a specialist has been linked to better knowledge in one’s area of speciality. Afhami et al. [30] confirmed that midwives with higher qualifications can provide better quality work than their counterparts as they are more knowledgeable in the area in which they specialised.

Maternal unit protocols have been prepared for the guidance of health workers such as doctors and midwives who provide obstetric, surgical and anaesthetic services for pregnant women in district clinics, health centres and district hospitals where specialist services are not always available [31]. The protocols provide a practical approach for primary health care to manage pregnancy, labour and delivery in South Africa with the ultimate aim of reducing maternal and perinatal mortality [32] and midwives are expected to utilise them in managing obstetric emergencies and complications. While many respondents in this study admitted to the protocols’ availability in their hospitals, up to 20% of the responded that the protocols were “sometimes”, “hardly ever” or “never” available, suggesting that such respondents were operating in maternity units which disregarded the protocols in their practices and this could be a contributory factor to the problem of perinatal mortality.

Furthermore, the fact that less than half of the respondents indicated that they always make use of the protocols, though they agreed that it is useful for managing obstetrics complications suggests that utilisation of the protocols was not being enforced consistently in the maternity wards. Whenever protocols were available, they were used as decision-making aids, especially when they were simple handy tools and in situations where providers were unsure what their next step in management should be [33]. Schack et al. [34] also confirmed that the implementation of protocols by midwives and doctors is beneficial to women who had complications during labour. Failure to have and/or follow standard protocols at primary and secondary care levels is one of the common problems related to poor perinatal outcomes.

This study also revealed that many respondents usually receive support from the hospital management with respect to interpretation and utilization of the protocols. Good access to support from the management has been linked to a better understanding and utilisation of maternity unit protocols [35].

The strong relationship between age, specialty and the work experience of participants with exposure to obstetrics training skills demonstrated in this study suggests an ongoing training schedule in the country for midwives. Application of the principles and skills learnt during such trainings is hoped to positively affect perinatal outcomes in the facilities [19,20]. These results also suggested that knowledge of HBB skills possessed by midwives could be improved by targeting midwives with a Diploma in General Nursing and Midwifery and encouraging them to participate in future HBB skills training workshops. However, the weak relationship between specialty qualifications and HBB training suggests that a lack of HBB skills in midwives was not constant across all specialty qualification categories, therefore, midwives who do not possess any specialised qualifications can also be encouraged to actively participate in HBB skills training workshops.

## 5. Conclusions

This study concluded that obstetrics skills are essential for midwives to be able to manage obstetric complications and reduce neonatal mortality in low- and middle-income countries. However, maternity unit protocols are not always utilised when managing obstetric complications in the hospitals selected for this study. We recommend that young or newly employed midwives should be encouraged to attend the training programmes and be made familiar with the protocols in obstetric emergencies. This could be achieved through regular in-service trainings on the consistent utilization of maternal unit protocols and the training should be made compulsory for all midwives. Public hospital authorities should take it upon themselves to monitor and encourage midwives to implement the use of the protocols and skills learnt when managing obstetric emergencies. We also recommend that future studies should look into the patient’s (maternal) factors that are associated with perinatal mortality, such that through a combination of efforts from healthcare providers and mothers, perinatal health can be further improved.

## Figures and Tables

**Table 1:** The sampling frame.

Hospital Code	Total No. of Midwives	Estimated No. of Deliveries per Month	Comment
Hospital A	65	475	Included
Hospital B	32	448	Included
Hospital C	47	388	Included
Hospital D	23	347	Excluded
Hospital F	24	303	Excluded
Hospital E	20	167	Excluded
Hospital G	20	126	Excluded
Total	231	2254	
Total included	144	1311	

**Table 2:** Socio-demographic characteristics of the respondents.

Socio-Demographic Characteristics		Frequency (*n*)	Percentage (%)
Gender	Male	2	1.9
	Female	103	98.1
	Total	105	100
Age (in years)	20–29	10	9.7
	30–39	15	14.6
	40–59	76	73.8
	>60	2	1.9
	Total	103	100
Nursing qualification	Diploma in midwifery	67	63.8
	Diploma in General nursing, Community, Psychiatry and Midwifery	14	13.3
	Degree in General nursing, Community, Psychiatry, and Midwifery	24	22.9
	Total	105	100
Specialty qualifications	Diploma in Advanced Midwifery and Neonatology	18	17.1
	Diploma in Neonatal Intensive Care Nursing	1	1
	Degree in Neonatal Intensive Care Nursing	2	1.9
	No specialty	84	80.0
	Total	105	100
Years of experience	2–10	29	23.2
	11–20	26	20.8
	21–30	30	24.0
	31–40	20	16.0
	Total	125	100

## Data Availability

The data analysed in this study can be obtained from the first author upon reasonable requests.

## References

[1] Pasha O., McClure E.M., Wright L.L., Saleem S., Goudar S.S., Chomba E., Patel A., Esamai F., Garces A., Althabe F. (2013). A combined community- and facility-based approach to improve pregnancy outcomes in low-resource settings: A Global Network cluster randomized trial. BMC Med..

[2] Akombi B.J., Renzaho A.M. (2019). Perinatal Mortality in Sub-Saharan Africa: A Meta-Analysis of Demographic and Health Surveys. Ann. Glob. Health.

[3] Debelew G.T. (2020). Magnitude and Determinants of Perinatal Mortality in Southwest Ethiopia. J. Pregnancy.

[4] MacDorman M.F., Mathews T.J., Mohangoo A.D., Zeitlin J. (2014). International comparisons of infant mortality and related factors: United States and Europe. Natl. Vital Stat. Rep..

[5] Ntenda P.A.M., Chuang K.Y., Tiruneh F.N., Chuang Y.C. (2014). Factors Associated with Infant Mortality in Malawi. J. Exp. Clin. Med..

[6] Requejo J.H., Bryce J., Barros A.J., Berman P., Bhutta Z., Chopra M., Daelmans B., De Francisco A., Lawn J., Maliqi B. (2015). Countdown to 2015 and beyond: Fulfilling the health agenda for women and children. Lancet.

[7] Statistics South Africa (2018). Perinatal Deaths in South Africa 2016. http://www.statssa.gov.za/publications/P03094/P030942016.pdf.

[8] Ezechi O.C., David A.N., Ezechi O.C., Odberg-Petterson K. (2012). Overview of Global Perinatal Mortality. Perinatal Mortality.

[9] Lawn J.E., Blencowe H., Waiswa P., Amouzou A., Mathers C., Hogan D., Flenady V., Frøen J.F., Qureshi Z.U., Calderwood C. (2016). Stillbirths: Rates, risk factors, and acceleration towards 2030. Lancet.

[10] Aune D., Saugstad O.D., Henriksen T., Tonstad S. (2014). Maternal body mass index and the risk of fetal death, stillbirth, and infant death: A systematic review and meta-analysis. JAMA.

[11] Aminu M., Unkels R., Mdegela M., Utz B., Adaji S., Den Broek N. (2014). Causes of and factors associated with stillbirth in low-and middle-income countries: A systematic literature review. BJOG Int. J. Obstet. Gynaecol..

[12] Liu L., Oza S., Hogan D., Perin J., Rudan I., Lawn J.E., Cousens S., Mathers C., Black R.E. (2015). Global, regional, and national causes of child mortality in 2000–13, with projections to inform post-2015 priorities: An updated systematic analysis. Lancet.

[13] Blencowe H., Cousens S., Jassir F.B., Say L., Chou D., Mathers C., Hogan D., Shiekh S., Qureshi Z.U., You D. (2016). National, regional, and worldwide estimates of stillbirth rates in 2015, with trends from 2000: A systematic analysis. Lancet Glob. Health.

[14] World Health Organization (2015). Global Health Observatory (GHO) Data 2015. world-health-statistics-2015.pdf.

[15] Maaya K.S., Gilmour S., Ota E., Shibuya K. (2017). Trends in perinatal mortality and its risk factors in Japan: Analysis of vital registration data, 1979–2010. Sci. Rep..

[16] Shukla V.V., Eggleston B., Ambalavanan N., McClure E.M., Mwenechanya M., Chomba E., Bose C., Bauserman M., Tshefu A., Goudar S.S. (2020). Predictive modeling for perinatal mortality in resource-limited settings. JAMA Netw. Open.

[17] Central Statistical Office (CSO) (2007). Zambia Demographic and Health Survey.

[18] Semrau K.E.A., Hirschhorn L.R., Marx Delaney M., Singh V.P., Saurastri R., Sharma N., Tuller D.E., Firestone R., Lipsitz S., Dhingra-Kumar N. (2017). Outcomes of a coaching-based WHO Safe Childbirth Checklist Program in India. N. Engl. J. Med..

[19] Patel A., Khatib M.N., Kurhe K., Bhargava S., Bang A. (2017). Impact of neonatal resuscitation trainings on neonatal and perinatal mortality: A systematic review and meta-analysis. BMJ Paediatr. Open..

[20] Versantvoort J.M.D., Kleinhout M.Y., Ockhuijsen H.D.L., Bloemenkamp K., de Vries W.B., van den Hoogen A. (2020). Helping Babies Breathe and its effects on intrapartum-related stillbirths and neonatal mortality in low-resource settings: A systematic review. Arch. Dis. Child..

[21] Chaka E.E., Mekurie M., Abdurahman A.A., Parsaeian M., Majdzadeh R. (2019). Association between place of delivery for pregnant mothers and neonatal mortality: A systematic review and meta-analysis. Eur. J. Public Health.

[22] South African National Department of Health (2021). South African National Department of Health Maternal, Perinatal, and Neonatal Health Policy. https://www.knowledgehub.org.za/system/files/elibdownloads/2021-06/SAMPNHPolicy23-6-2021signedWebViewv2.pdf.

[23] Kassebaum N.J. (2021). Global, regional, and national progress towards Sustainable Development Goal 3.2 for neonatal and child health: All-cause and cause-specific mortality findings from the Global Burden of Disease Study 2019. Lancet.

[24] Vhembe District Municipality (2020). 34/52 Profile and Analysis District Development Model. https://www.cogta.gov.za/ddm/wp-content/uploads/2020/11/Vhembe-October-2020.pdf.

[25] Vhembe District Municipality 2018/19-2021/22 IDP Review and Amendment. http://vhembe.gov.za/media/content/documents/2019/9/o_1dnpb8nfs1je1nvhe4314a21tks8.pdf?filename=VHEMBE%20DISTRICT%20MUNICIPALITY%20IDP%20REVIEW%20AND%20AMANDEMENT%202018%20l%202019-2021%20l%202022.pdf.

[26] Seawright J. (2016). The Case for Selecting Cases That Are Deviant or Extreme on the Independent Variable. Sociol. Methods Res..

[27] Etikan I., Musa S.A., Alkassim R.S. (2016). Comparison of Convenience Sampling and Purposive Sampling. Am. J. Theor. Appl. Stat..

[28] Cottingham M. (2014). Recruiting men, constructing manhood: How healthcare organizations mobilize masculinities as nursing recruitment strategy. Gend. Soc..

[29] Achora S. (2016). Conflicting images: Experiences of male nurses in a Uganda’s hospital. Int. J. Afr. Nurs. Sci..

[30] Afhami N., Bahadoran P., Taleghani H.R., Nekuei N. (2016). The knowledge and attitudes of midwives regarding legal and religious commandments on induced abortion and their relationship with some demographic characteristics. Iran. J. Nurs. Midwifery Res..

[31] National Department of Health, Republic of South Africa (2015). Guidelines for Maternity Care in South Africa: A manual for Clinics, Community Health Centres and District Hospitals. http://www.hst.org.za/publications/NonHST%20Publications/Maternal%20Care%20Guidelin.

[32] Malherbe H.L., Woods D.L., Aldous C., Christianson A.L. (2016). Review of the 2015 Guidelines for Maternity Care with relevance to congenital disorders. S. Afr. Med. J..

[33] Oduro-Mensah E., Kwamie A., Antwi E., Bamfo S.A., Bainson H.M., Marfo B., Coleman M.A., Grobbee D.E., Agyepong I.A. (2013). Care Decision Making of Frontline Providers of Maternal and New-born Health Services in the Greater Accra Region of Ghana. PLoS ONE.

[34] Schack S.M., Elyas A., Brew G., Pettersson K.O. (2014). Experiencing challenges when implementing Active Management of Third Stage of Labor (AMTSL): A qualitative study with midwives in Accra, Ghana. BMC Pregnancy Childbirth.

[35] Ngongo C., Christie K., Holden J., Ford C. (2013). Striving for excellence: Nurturing midwives’ skills in Freetown, Sierra Leone. Midwivery.

